# Efficacy of BCMA CAR-T cell therapy and subsequent strategies in refractory and relapsed plasma cell leukemia: a retrospective cohort study

**DOI:** 10.3389/fimmu.2026.1756209

**Published:** 2026-02-05

**Authors:** Yuelu Guo, Lixia Ma, Fan Yang, Zhonghua Fu, Danyang Li, Rui Liu, Miaomiao Cao, Bian Wei, Yimeng Dou, Biping Deng, Shilin Gan, Alex H. Chang, Xiaoyan Ke, Kai Hu

**Affiliations:** 1 Department of Lymphoma and Myeloma Research Center, Beijing Gobroad Hospital, Beijing, China; 2 Cytology Laboratory, Beijing GoBroad Boren Hospital, Beijing, China; 3 Department of Biostatistics, GoBroad Research Center, Beijing, China; 4 Engineering Research Center of Gene Technology, Ministry of Education, Institute of Genetics, School of Life Sciences, Fudan University, Shanghai, China; 5 Shanghai YaKe Biotechnology Ltd., Shanghai, China

**Keywords:** allogeneic hematopoietic stem cell transplantation, BCMA CAR-T, cytokine release syndrome, immune effector cell-associated neurotoxicity syndrome, refractory and relapsed plasma cell leukemia

## Abstract

**Background:**

Plasma cell leukemia (PCL) is a rare and aggressive hematological malignancy. The long-term prognosis of relapsed/refractory plasma cell leukemia (R/R PCL) remains poor, and few treatment options are available for patients with triple-refractory disease. Chimeric antigen receptor (CAR)-T cell therapy targeting the B-cell maturation antigen (BCMA) has shown promise, though its long-term efficacy and optimal subsequent strategies remain to be fully elucidated.

**Methods:**

This retrospective study analyzed the efficacy and safety of BCMA CAR-T therapy in 12 patients with triple-class R/R PCL. Patients were stratified into consolidation (Group 1, allo-HSCT within 3 months post-CAR-T) and non-consolidation (Group 2, no allo-HSCT within 3 months post-CAR-T) groups, with survival outcomes compared between cohorts.

**Results:**

The overall response rate following BCMA-CAR-T cell therapy was 75% (9/12); four patients achieved partial response, four achieved very good partial response, and one patient had complete response. Grade 3–4 cytopenia were universally observed, while 83.3% (10/12)of the patients presented with mild (grade 1-2) cytokine release syndrome. The median progression free survival (PFS) was 8.9 months (95% CI: 4.6, not reached). The 1-year PFS rate was 33.3% (95% CI: 7.8–62.3), and the 2-year PFS rate was 22.2% (95% CI: 3.4–51.3). The median overall survival (OS) was 15.5 months (95% CI: 5.7, not reached). The 1-year OS rate was 55.6% (95% CI: 20.4–80.5), and the 2-year OS rate was 22.2% (95% CI: 3.4–51.3). furthermore, two of the four patients who underwent consolidation therapy showed long-term survival with stringent complete response.

**Conclusions:**

BCMA-CAR-T therapy confers short-term remission and survival benefits in relapsed/refractory plasma cell leukemia (R/R PCL). However, the definitive value of allogeneic hematopoietic stem cell transplantation (allo-HSCT) awaits validation in large-sample prospective studies.

## Introduction

Primary plasma cell leukemia (PCL) is a rare, highly aggressive clonal plasma cell neoplasm. The International Myeloma Working Group (IMWG) redefined the diagnostic criterion for PCL as ≥5% plasma cells in peripheral blood in 2021 (1–4). Secondary PCL (sPCL) is characterized by leukemic-phase evolution after post-treatment relapse of multiple myeloma (5). Both pPCL and sPCL have high proliferative indices and are linked to poor prognosis (6–8). Retrospective studies indicate that despite novel agent therapy, newly diagnosed pPCL has a median progression-free survival (PFS) of only 5.5 months and median overall survival (OS) of 18.1 months (5). sPCL carries an even worse prognosis, with a median OS of 4.2 months and 1-year OS rate of merely 19% (9).

Therapeutic options for R/R PCL remain scarce and prognosis dismal (10, 11). Although studies indicate survival benefit from autologous stem-cell transplantation in pPCL (12, 13), therapeutic choices are extremely limited for post-transplant relapses, particularly triple-class refractory cases. In recent years, CAR-T-cell therapy targeting the B cell maturation antigen (BCMA) has achieved high overall response rates (ORR) of 73-98% for relapsed/refractory multiple myeloma (RRMM) (14–16). However, only a few reports are available on the use of BCMA-CAR-T cell therapy for R/R PCL. A recent study documented an ORR of 90% at day 30 and 86% at day 90 among 30 PCL patients treated with idecabtagene vicleucel (ide-cel) or ciltacabtagene autoleucel (cilta-cel); However, there remains a research gap regarding the impact of post-treatment strategies on long-term prognosis in patients with R/R PCL following BCMA CAR-T cell therapy. (17–19). Studies show that consolidation with allogeneic hematopoietic stem cell transplantation (allo-HSCT) can prolong the survival of patients with refractory/relapsed B cell lymphoblastic leukemia (B-ALL) who achieve remission after CD19-CAR-T cell therapy (20, 21) Furthermore, allo-HSCT has been shown to confer survival benefits in PCL patients (22, 23). This retrospective study aimed to evaluate the safety and efficacy of BCMA CAR-T cell therapy in R/R PCL patients, and assess the impact of subsequent therapies post CAR-T cell therapy on long-term outcomes.

## Methods

PCL patients who provided written informed consent for BCMA-CAR-T cell therapy at our center between May 2021 and February 2025 were retrospectively identified. The baseline diagnosis of primary or secondary PCL, clinical characteristics, cytogenetic profiles, and mutational landscapes were extracted from the hospital electronic medical records. High-risk cytogenetic abnormalities (HRCAs) were defined according to the 2021 IMWG criteria for high-risk multiple myeloma (HRMM): del(17p) with a clonal burden >20% and/or TP53 mutation; IgH translocations t(4;14), t(14;16) or t(14;20) accompanied by 1q gain and/or del(1p32); mono-allelic del(1p32) co-existing with 1q gain; or bi-allelic del(1p32) (24).

The details of pre-infusion bridging chemotherapy protocol, including the specific time, drug dosages were also recorded. The process by which CAR-T cells destroy tumors: Firstly, peripheral blood mononuclear cells (PBMC) were collected. After *in vitro* sorting and activation of T cells, lentiviral vector transduction and CAR-T cell expansion were carried out. After 7–10 days of cell culture, the cells were collected for quality testing. Once they met the release standards, they were reinfused into the patient’s body. Structural design of Chimeric antigen receptors (CARS):The schematic diagram shows the second-generation CAR. Besides the CD3 ζ signal transduction domain within the cell, there is also a costimulatory signal transduction domain 4-1BB.

The efficacy of CAR-T cell therapy was assessed according to the 2016 revised efficacy criteria of IMWG (4). In our study, CRS and ICANS following BCMA CAR-T therapy were graded and managed in adherence to the ASTCT consensus criteria, which ensured standardized assessment of treatment-related toxicities and facilitated comparison with findings from other clinical studies.(ASTCT, now known as the American Society for Blood and Marrow Transplantation, or ASBMT) (25).CAR-T cell expansion and cytokine levels were assessed on days 3, 7, 11, 15, 21, and 28 following BCMA-CAR-T cell infusion. Hematological toxicity and organ toxicity were graded by applying the Common Terminology Criteria (CTCAE) version 5.0.

Patients who received allogeneic hematopoietic stem cell transplantation (allo-HSCT) within 3 months after BCMA CAR-T cell therapy were assigned to the Group1(consolidation therapy group), whereas those who did not receive allo-HSCT were categorized into the Group2(non-consolidation therapy group).In the non-consolidation group (n=5), Pt.04, Pt.08, and Pt.09 received pomalidomide maintenance;Pt.06 did not due to pneumonia. And the disease status and survival outcomes of the two groups were compared.

**Figure 1:**
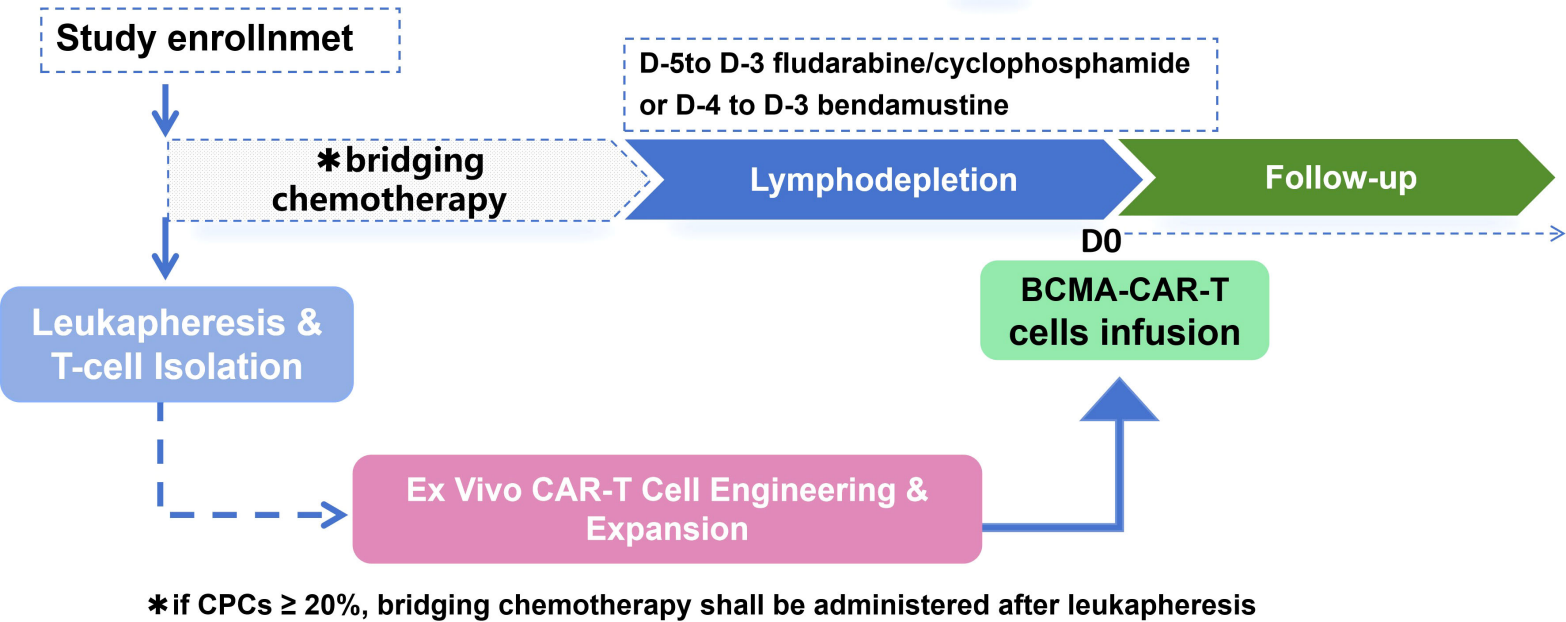
Flow chart of retrospective study on BCMA CAR-T therapy for R/R PCL.

PFS was defined as the time from the start of CAR-T cell infusion to disease progression or death from any cause, or follow-up up to date. OS was defined as the time from the start of CAR-T cell infusion to the date of death from any cause or the follow-up cutoff date. For the surviving patients, the data was truncated at the last follow-up visit or at the end of the study. Please refer to **Figure 1** for the subsequent treatment process of enrolled patients.

Survival curves were generated using Kaplan-Meier estimates with GraphPad Prism v9.0. Hazard ratios (HRs) and 95% confidence intervals (CIs) were calculated for PFS/OS. Due to the small cohort size, multivariate analysis was not feasible; however, subgroup analyses (e.g., consolidation therapy group vs. non-consolidation therapy group) were performed using log-rank tests. Patients were followed up every 3 months after the end of treatment to assess disease status and record survival. For patients lost to follow-up, the survival status was updated by phone, mail, or medical record inquiry.

## Result

### Baseline characteristics and bridging therapy

Twelve patients who presented to the Beijing GoBroad Hospital met the definition of PCL during the study period. Only three patients were older than 65 years old, and four patients presented with pPCL. Ten patients harbored HRCAs, and eight patients had M protein level above 20 g/L. All patients had triple-class refractoriness to immunomodulatory drugs, proteasome inhibitors (PI) and daratumumab, and five patients had previously undergone autologous transplantation. The clinical characteristics of the patients are summarized in **Table 1**.

**Table 1:** Summary of CRS, ICANS, and hematologic toxicity following bcma CAR-T cell infusion.

Case/characteristic	Age	Gender	ECOG	pPCL or sPCL	Paraprotein characterization	R-ISS stage	HRCAs	FISH or NGS	EMD	CPC	Bone marrow blast	M protein (g/l)	dFLC (mg/l)	K/L ratio	BCMA expression rate	Number of previous lines of treatment	drug refractoriness before treatment
Pt.1	65	male	4	pPCL	IgA	III	Y	t(14,16) p53mutation CKS1B RB1	0	42%	84%	36.3	9.3	2.788	84.72%	3	Lenalidomide, Pomalidomid Bortezomib Daratumumab
Pt.2	71	female	1	pPCL	IgG	III	Y	t(4,14) 1q21amplification p53mutation D13S319 RB1	0	33%	52.5%	31.9	9	1.783	81.25%	3	Lenalidomide, Pomalidomid Bortezomib Daratumumab
Pt03.	50	male	3	sPCL	IgG	III	Y	1q21amplification del(17p) t(4;14) RB1+ D13S319+	1(central nervous system)	5.3%	42%	1.1	>4369.1	<0.001	80%	3	Bortezomib Pomalidomid Daratumumab Auto-SCT
Pt04.	59	male	1	sPCL	IgA	III	Y	1q21amplificatio; D13S319+ RB1+ t(4;14)	1(inguinal soft tissue)	6%	84.5%	26.1	7.4	1.673	70%	5	Bortezomib Lenalidomide Pomalidomid
Pt.5	44	male	1	pPCL	IgG	III	NA	1q21amplification RB1 IgHtranslocation	0	6%	91%	38.3	102.091	0.009	93.77%	2	Lenalidomide, Pomalidomid Bortezomib Daratumumab
Pt.6	68	male	1	sPCL	Light chain only kappa	III	NA	NA	1(paravertebral mass)	16%	91.5%	0	600.88	537.5	63.47%	4	Lenalidomide, Pomalidomid Bortezomib Carfilzomib Daratumumab
Pt07	62	male	1	sPCL	Light chain only kappa	III	Y	IgHtranslocation D13S319 RB1 1q21amplificatio; p53mutation	0	11%	75%	0	>4220	>845	80%	4	Lenalidomide, Pomalidomid Bortezomib Daratumumab
Pt.08	53	male	1	sPCL	IgG	II	Y	t(4,14) del(17p) 1q21amplification D13S319 RB1	0	10%	32.5%	42.4	1578.76	1274.194	85.37%	7	Lenalidomide Bortezomib Carfilzomib Daratumumab Auto-SCT
Pt.09	43	male	1	sPCL	IgA	III	Y	del(17p) 1q21amplification p53mutation	0	6%	9.5%	48.3	>4294.18	<0.001	99.73%	5	Lenalidomide Bortezomib Daratumumab
Pt.10	44	male	1	sPCL	IgG	II	Y	1q21amplification del(17p) p53mutation D13S319+ RB1+ 8q21+	0	35%	68.5%	40.2	632.08	0.009	16.93%	1	Lenalidomide Bortezomib Daratumumab Auto-SCT
Pt.11	44	male	1	pPCL	IgG	III	Y	RB1+, del(1p32.3) 1q21amplification	1(duodenal mass)	7%	71.5%	3.9	1.08	0.74	90%	3	Lenalidomide, Pomalidomid Bortezomib Carfilzomib Daratumumab Auto-SCT
Pt.12	35	female	1	sPCL	IgG	II	Y	t(14,16) 1q21amplification p53mutation	0	28%	69%	39.9	209.55	0.032	80.24%	7	Lenalidomide Bortezomib Daratumumab Auto-SCT

ECOG, Eastern Cooperative Oncology Group R-ISS, Revised International Staging System;EMD, Extramedullary Disease;HRCA, high-risk cytogenetic abnormalities; CPC, Circulating Plasma Cells; dFLC, Differential Free Light Chains;K/L, Kappa/Lambda Ratio; pPCL, primary plasma cell leukemia; sPCL, Secondary plasma cell leukemia;NA, Not Available

Patients with CPCs ≥ 20% received bridging chemotherapy after leukapheresis. The bridging chemotherapy for 4 patients were as follows: Pt.01 received bendamustine 100 mg on days 1-2, pomalidomide 4 mg on days 1-14, and dexamethasone 20 mg on days 1-2, 8-9, 15-16; Pt. 02 was administered daratumumab 800 mg on days 1, 8, 15, and 22, bortezomib 1.7 mg on days 1, 4, 8, and 11, pomalidomide 4 mg once daily (qd) on days 1-14, selinexor 40 mg twice weekly, and dexamethasone 40 mg on days 1, 8, 15, and 22; Pt. 10 received selinexor 20 mg twice weekly, pomalidomide 4 mg qd, and dexamethasone 20 mg qd on days 1–4 and 8-12; Pt. 12 was given carfilzomib 60 mg on days 1-2, dexamethasone 20 mg on days 1-2, and pomalidomide 4 mg on days 1-7.

### CAR-T cell infusion and expansion kinetics

Lymphodepleting chemotherapy was administered on days −5/−4 to −3, with the regimen consisting of either fludarabine at a dose of 30 mg/m² for 3 days plus cyclophosphamide at 300 mg/m² for 3 days, or bendamustine at 90 mg/m² for 2 days. The dose of BCMA CAR-T cells ranged from 0.1 to 20.4 × 10^5^ cells/kg, with a median dose of 3.82 × 10^5^ cells/kg.

**Figure 2:**
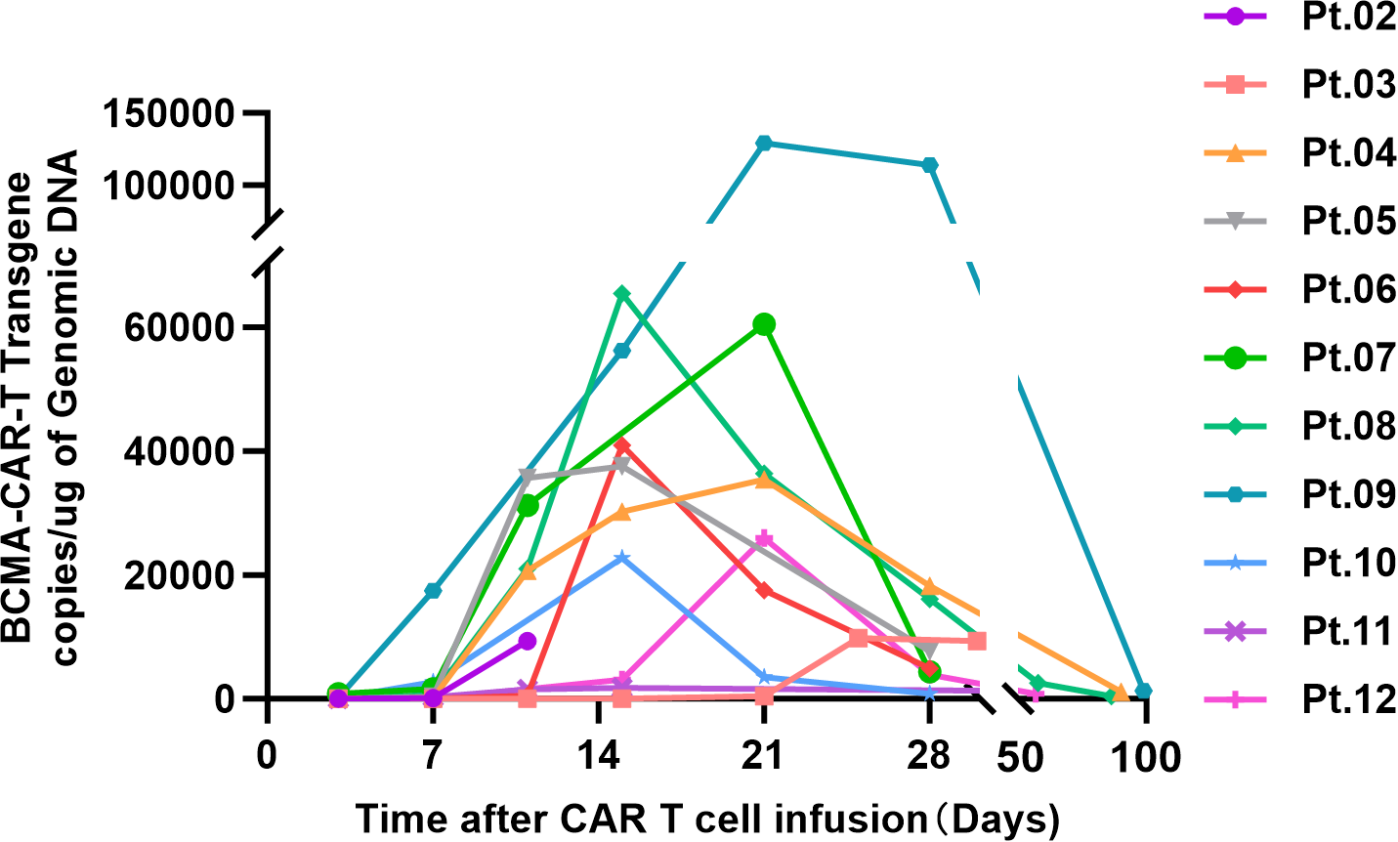
The vector copy number of the CAR-T cell per microgram of genomic DNA, measured by qPCR.

**Figure 3:**
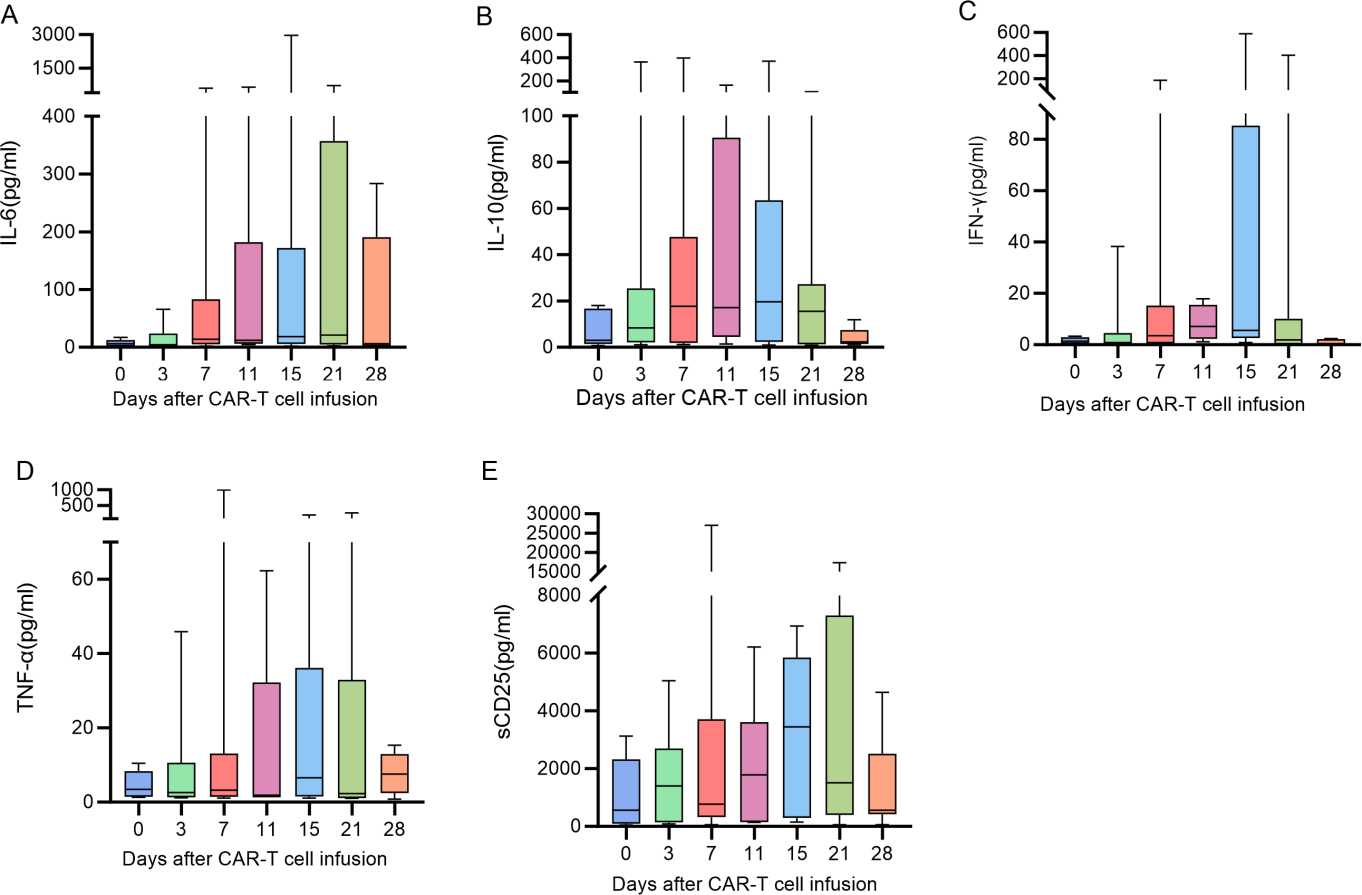
**(A–E)** The trend of cytokines one month after BCMA-CAR-T infusion.

The peak of CAR-T cell expansion was mostly detected at 7 to 14 days following CAR-T cell infusion (**Figure 2**).patients presented substantial interindividual differences in cytokine levels, and the majority of cytokine peaks emerged during the first to second week post-infusion (**Figures 3A–E**).

### Adverse events

The majority of patients (83.3%, 10/12) developed grade 1–2 CRS, and notably, no ICANS was observed. Severe hematologic toxicity was universal, with 100% of patients experiencing grade 3–4 anemia and 83.3% experiencing grade 3–4 neutropenia or thrombocytopenia (**Table 2**),Hematologic toxicities gradually resolved within 1–2 months after CAR-T cell infusion.

**Table 2:** Summary of CRS, ICANS, and hematologic toxicity following BCMA CAR-T cell infusion.

Case	CRS	ICANS	Anemia	Neutropenia	Thrombocytopenia
Pt.01	NA	NA	Grade3	Grade1	Grade4
Pt.02	NA	NA	Grade3	Grade4	Grade4
Pt.03	1	0	Grade4	Grade4	Grade4
Pt.04	1	0	Grade3	Grade4	Grade4
Pt.05	1	0	Grade3	Grade3	Grade2
Pt.06	2	0	Grade3	Grade4	Grade4
Pt.07	1	0	Grade3	Grade4	Grade4
Pt.08	1	0	Grade3	Grade4	Grade4
Pt.09	1	0	Grade3	Grade4	Grade4
Pt.10	1	0	Grade3	Grade4	Grade4
Pt.11	1	0	Grade4	Grade2	Grade1
Pt.12	2	0	Grade3	Grade4	Grade4

CRS, Cytokine Release Syndrome;ICANS, Immune Effector Cell-Associated Neurological Syndrome;NA = Not assessable (patient expired prior to CRS evaluation);Hematologic toxicities were graded according to the Common Terminology Criteria for Adverse Events (CTCAE) version 5.0.

Three patients died within 45 days after infusion. The details of their conditions are as follows:**Pt.01:**Pre-CAR-T infusion, CPCs rose to 70% with pre-existing cardiac insufficiency and atrial fibrillation history. Post-BCMA CAR-T infusion, the patient developed progressive deterioration manifesting as persistent fever, followed by heart failure, renal failure, gastrointestinal bleeding and hemorrhagic shock. Despite aggressive interventions (blood product transfusion, anti-infective therapy, moderate-dose glucocorticoids, targeted gastrointestinal bleeding management), clinical response was unsatisfactory, and the patient expired on day 3 post-infusion.

Pt.02:Post-bridging therapy, the patient’s CPCs decreased to 23% pre-CAR-T infusion but rebounded to 77% on day 7 post-infusion, Concurrently, grade 1 cytokine release syndrome (CRS) and fever developed on day 7. On day 8, the patient presented with gastrointestinal bleeding, hemorrhagic shock, grade 4 hematological toxicity, grade 4 gastrointestinal adverse events, and progressive grade 4 CRS. Despite aggressive interventions (non-invasive ventilation, vasoactive agents, fluid resuscitation, gastrointestinal bleeding management, glucocorticoid therapy), clinical response was unsatisfactory. The patient’s family opted for treatment withdrawal, and the patient expired subsequently.

Pt.03:CAR-T cell expansion was undetectable until 2 weeks post-infusion. Fever developed on day 16 without hypotension or hypoxemia, with only minimal CAR-T cells detected on the same day. On day 28, re-evaluation revealed serum free lambda light chain >4375 mg/L, urinary lambda light chain 1260.00 mg/L, and bone marrow plasma cell proportion 68.5%; additionally, the patient had progressive generalized pain, consistent with disease progression. The patient’s family opted for treatment withdrawal, and the patient was discharged before expiring subsequently.

### Efficacy assessment

**Figure 4:**
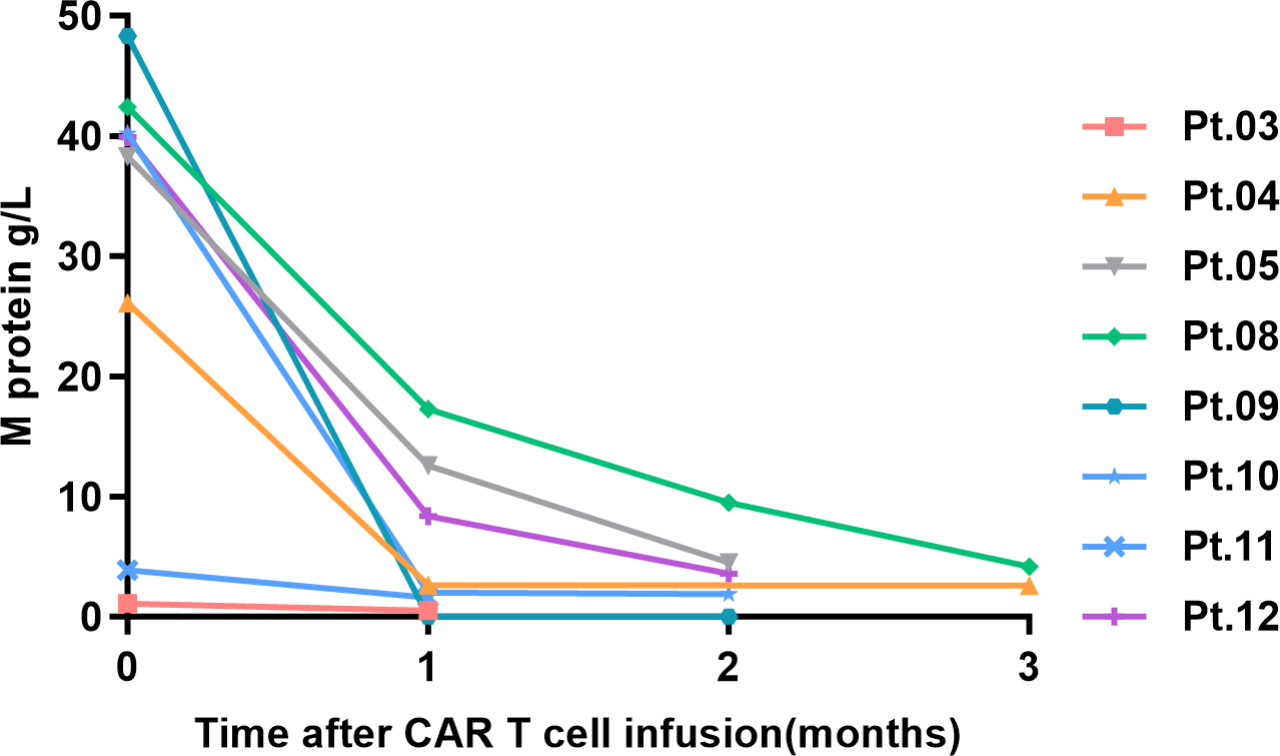
Serum M protein changes within 3 months post BCMA-CAR-T infusion.

The changes in M protein levels in the evaluable cases during the 3 months following BCMA-CAR-T cell infusion are shown in **Figure 4**. Measurable disease in Pt.06 and Pt.07 was monitored by serum free-light-chain (sFLC) assay. For Pt.06 the baseline dFLC was 600.88 mg/L, and fell to 4.96 mg/L, 4.96 mg/L and 0 mg/L at1, 2 and 3 months post-infusion respectively. For Pt.07, the baseline dFLC was >4200 mg/L, and decreased to 0.6 mg/L at 1 month and 10.3 mg/L at month 2 post-infusion.

The best ORR after BCMA-CAR-T cell therapy was 75% (9/12); four patients achieved partial response (PR), four achieved very good partial response (VGPR), and one had complete response (CR).

### Survival outcomes

**Figure 5:**
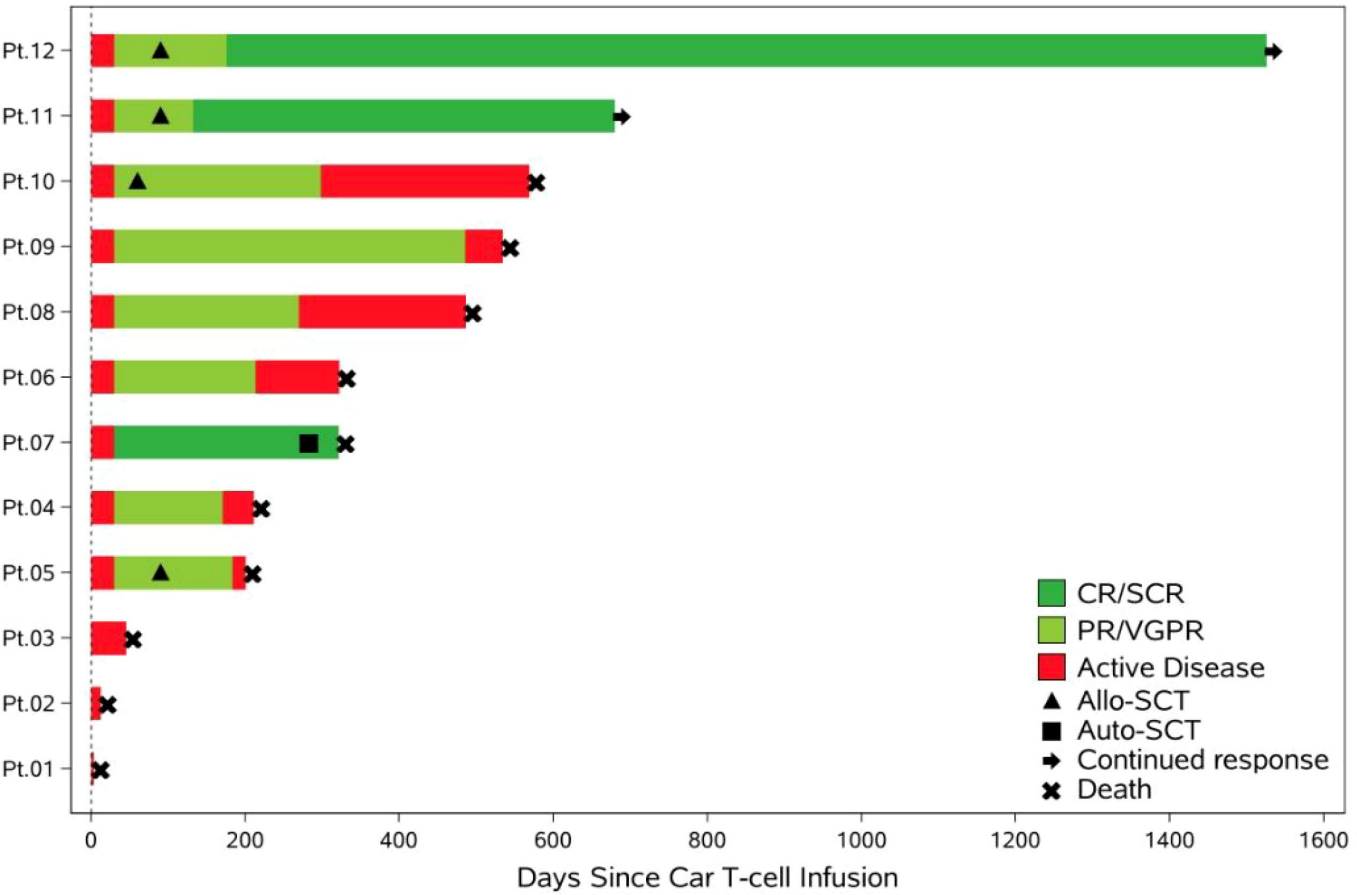
Swim-lane plot of post-treatment outcomes.

The survival after BCMA-CAR-T cell therapy have been summarized in **Figure 5**. Patients 12 and 11 remained in stringent complete remission (sCR) after BCMA-CART therapy following allogeneic stem cell transplantation (Allo-SCT), while all patients who did not receive Allo-SCT died.

The median time to recurrence for, **Pt.04, Pt.06**, **Pt.08**, and **Pt.09** was 211 days, with individual recurrence times of 455, 239, 183, and 140 days, respectively. Among these patients, **Pt.04** developed central nervous system (CNS) recurrence. Treatment with pegylated liposomal doxorubicin, bendamustine, bortezomib, and dexamethasone yielded suboptimal efficacy. **Pt.06** suffered from pulmonary fungal infection after BCMA-targeted CAR-T cell therapy. **Pt.06** together with **Pt.09** declined further antineoplastic treatment and opted for palliative care following disease recurrence.

**Pt.08**. The patient achieved initial remission post-BCMA-CAR-T therapy. However, disease recurrence occurred at 6 months due to intermittent blood transfusion support required for hematopoietic stem cell transplantation preparation. Despite allo-HSCT administration post-recurrence, the patient ultimately expired from cardiac arrest during the transplantation procedure.

**Pt.07**. The patient underwent autologous transplantation 8 months post-CAR-T infusion. One month post-transplant, severe pneumonia (mixed bacterial/fungal etiology), hypoxemia, and diarrhea developed. Despite non-invasive ventilation, antibacterial, and antifungal therapies, clinical response was unsatisfactory, and the patient ultimately succumbed to severe pneumonia.

The median follow-up time for this cohort of patients was 15.6 months. The median progression free survival (PFS) was 8.9 months (95% CI: 4.6, not reached). The 1-year PFS rate was 33.3% (95% CI: 7.8–62.3), and the 2-year PFS rate was 22.2% (95% CI: 3.4–51.3). The median overall survival (OS) was 15.5 months (95% CI: 5.7, not reached). The 1-year OS rate was 55.6% (95% CI: 20.4–80.5), and the 2-year OS rate was 22.2% (95% CI: 3.4–51.3).

**Figure 6:**
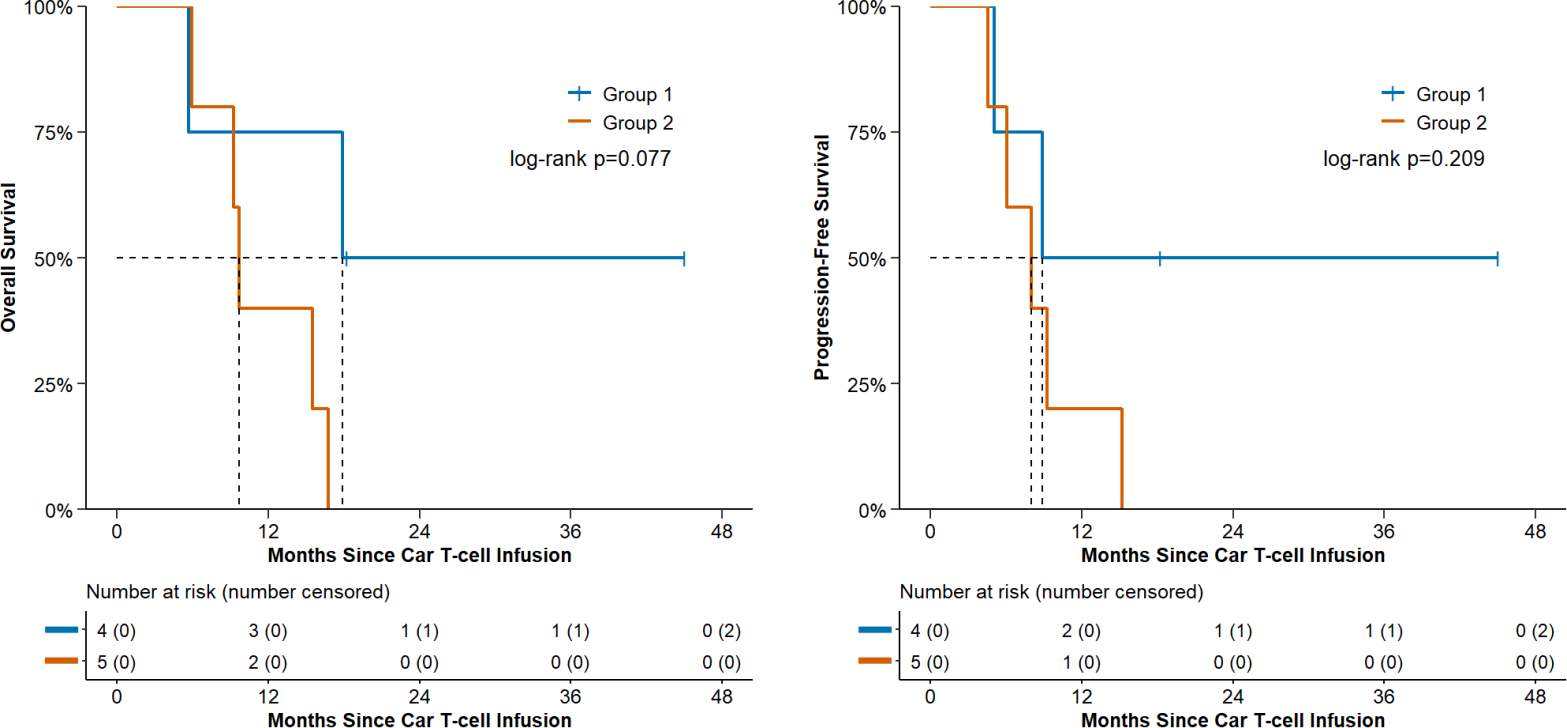
Progression-free survival and overall survival of patients in group 1 vs. group 2 post CAR-T cell infusion.

Patients receiving consolidation therapy had a 1-year overall survival (OS) rate of 50% (2/4), compared with 0% (0/5) in the non-consolidation group (P = 0.077). See **Figure 6** for details.

## Discussion

To date, published studies lack long-term survival data for BCMA-targeted CAR-T-treated patients. This retrospective study describes the clinical features and treatment courses of 12 R/R PCL patients, identifying marked heterogeneity in their long-term prognosis, which we discuss herein. Among patients with evaluable molecular genetic data, chromosome 17p abnormalities (mutations/deletions) were most common and consistent with published literature on plasma cell leukemia’s genomic landscape (26).

Pt. 01 died on day 3 post CAR-T infusion, with disease progression as the likely cause. For Pt. 02, whether death resulted from CRS or disease progression merits discussion: the patient had suboptimal bridging therapy response (pre-infusion CPC elevation; 77% CPC + fever on day 7; rising CAR-T copy number vs. day 3), deteriorated rapidly on day 8 (gastrointestinal bleeding, hemorrhagic shock) and died after treatment withdrawal. High pre-infusion tumor burden was identified as a severe CRS risk factor in the CARTITUDE trial. (27). Additionally, published evidence indicates bridging therapy improves prognosis and controls adverse events in CNS-involved relapsed/refractory multiple myeloma (RRMM) patients. (28), However, no definite benefit of pre-CAR-T bridging therapy has been demonstrated in B-cell non-Hodgkin lymphoma (B-NHL) patients. (29). No reports have addressed bridging therapy use in R/R PCL. For Pts. 10 and 12, bridging therapy reduced CPCs from 33%/28% to 6%/11% with grade 1/2 CRS, respectively. Thus, high tumor burden patients unresponsive to bridging therapy may not be suitable for CAR-T. In contrast, safe/effective bridging may improve subsequent BCMA-CAR-T response, warranting optimal tumor debulking regimen exploration. However, high-dose cytotoxic bridging may impair post-BCMA-CAR-T hematopoietic recovery. (30).

Marked myelosuppression was observed in this cohort: all had grade 3–4 anemia, 83.3% (10/12) grade 3–4 neutropenia/thrombocytopenia. Notably, grade 4 myelosuppression incidence was significantly higher than prior reports in BCMA-CAR-T RRMM. (31, 32). This discrepancy may be due to prior multiple lines of therapy and higher intramedullary tumor burden in our cohort. Further studies are needed to reduce risks and improve BCMA-CAR-T safety in R/R PCL.

Among patients with evaluable efficacy post-BCMA-CAR-T, ORR was 75% (9/12), lower than reported 30 day (90%) and 90 day (86%) ORR of ide-cel/cilta-cel in plasma cell leukemia. This discrepancy may be due to a higher sPCL proportion in our cohort (66.7%, 8/12) vs. ide-cel (53%, 10/19) and cilta-cel (7%, 1/15) cohorts. (17). Its ORR was comparable to BCMA-CAR-T reports in RRMM, whereas CR rate was significantly lower (only 1 patient achieving CR). (16, 32–34). This finding indicates BCMA-CAR-T yields only limited short-term remission depth in this cohort.

Relapse in PCL is multifactorial. Pt.04’s CNS recurrence underscores the sanctuary site effect, while PCL’s genomic instability promotes clonal evolution and antigen escape. High tumor burden may also induce CAR-T exhaustion via antigen overload or signal dilution, compounded by potentially impaired T-cell fitness in heavily pretreated patients. Combination strategies, including bispecific antibodies or GPRC5D-targeted therapy, warrant further investigation to address these resistance mechanisms.

Among 9 patients with evaluable survival outcomes, mPFS was 8.9 months (95% CI, 4.6–NE) with 33.3% 1-year PFS (95% CI, 7.8%–62.3%), and mOS was 15.5 months (95% CI, 5.7–NE) with 55.6% 1-year OS (95% CI, 20.4%–80.5%). These 1-year outcomes were superior to prior BCMA-CAR-T data for plasma cell leukemia. (19, 35). These survival outcomes were comparable to the reported mPFS/mOS of ide-cel/cilta-cel in plasma cell leukemia (9/13 months, respectively). (17).

No significant PFS/OS differences were observed between the consolidation and non-consolidation groups. A higher 1-year OS trend was noted in the consolidation group (50%, 2/4 vs. 0%, 5/5, P = 0.077, Fisher’s exact test), yet not statistically significant. Moreover, small sample size precluded baseline matching/adjustment between groups; additionally, Pt.07 in the non-consolidation group died of non-relapse causes. Thus, these findings are insufficient to confirm a consolidation therapy survival advantage.

Analysis of two long-term survivors (Pt.11, Pt.12) revealed both had an ECOG performance status score of 1: Pt.11 (44 years, 3 prior lines of therapy); Pt.12 (35 years, cohort’s youngest, 7 prior lines of therapy). KPD bridging therapy induced marked CPC reduction in Pt.12, indicating effective tumor debulking. Both achieved ≥PR post-BCMA-CAR-T and underwent allo-HSCT within 3 months, attaining OS of 18.2 and 45 months, respectively, with ongoing sCR at writing. Given the median time to recurrence was 7 months post-BCMA-CAR-T infusion, we hypothesize optimal sequential allo-HSCT timing may be within 6 months of achieving ≥PR, particularly for young patients without cardiorenal comorbidities. This hypothesis warrants validation in larger prospective datasets.

As a non-randomized retrospective study, this research has inherent limitations: small sample size and high patient heterogeneity caused baseline group imbalances. Future studies should expand sample size and design well-powered prospective trials to identify optimal therapeutic strategies for this population.

## Conclusions

BCMA CAR-T therapy can yield certain short-term remission and survival benefits in patients with R/R PCL.allogeneic hematopoietic stem cell transplantation (allo-HSCT) definite value still requires validation in large-sample prospective studies. In the future, further exploration of safe and effective bridging tumor debulking regimens and optimal consolidation treatment procedures is needed to improve efficacy and safety.

## Data Availability

The raw data supporting the conclusions of this article will be made available by the authors, without undue reservation.
